# Editorial: The immunology of the male genital tract

**DOI:** 10.3389/fimmu.2022.1042468

**Published:** 2022-10-17

**Authors:** Kenneth S. K. Tung, Daishu Han, Yong-Gang Duan

**Affiliations:** ^1^ Department of Pathology, Beirne B. Carter Center of Immunology, University of Virginia, Charlottesville, VA, United States; ^2^ School of Basic Medicine, Peking Union Medical College, Beijing, China; ^3^ Shenzhen Key Laboratory of Fertility Regulation, Center of Assisted Reproduction and Embryology, University of Hong Kong – Shenzhen Hospital, Shenzhen, China

**Keywords:** editorial, male reproductive immunology, chronic epididymitis, experimental autoimmune orchitis (EAO), autoimmunity

Research on organ-specific autoimmune disease generally covers both systemic and local regulations. In contrast, studies on the testis are focused mainly on local mechanisms. This is because of the general belief that haploid germ cell-specific antigens (Ag) are sequestered within the seminiferous tubule (SFT) by the blood–testis barrier (BTB) and do not communicate with the immune system and that the late ontogeny of haploid germ cell Ag precludes neonatal tolerance induction. However, these arguments are not evidence-based, and recent findings indicate they are not valid.

As observed by Harakal et al., Ag sequestration applies only to some sperm Ag. Other haploid sperm Ag continuously egress the normal SFT. The physiological sperm Ag lactate dehydrogenase 3 (LDH3) enters the residual body (RB), egresses into the testis interstitial space, and forms immune complexes with circulating antibodies (Ab). LDH3 first egresses at postnatal week 5 when RB formation begins. Ag-specific systemic tolerance to LDH3 is documented in male wild-type (WT) mice, but not in male LDH3 -/- mice or Treg-depleted male WT mice. When Tregs are depleted from adult mice expressing the transgenic Foxp3-diphtheria toxin receptor, they spontaneously develop LDH3 Ab and severe autoimmune orchitis (AO), and both are preventable with Treg from WT male donors. Therefore, the egress of the sperm Ag from the SFT beyond the neonatal period is protected by Treg-dependent systemic tolerance. A similar mechanism applies to autoantigens in mouse stomachs, ovaries, prostates, and lacrimal glands (1–4). Although egress is documented herein only for LDH3, other investigators detected numerous rodent and human sperm proteins in the testis interstitial fluid and circulation *via* mass spectroscopy (5).

Normally sequestered sperm Ag are exemplified by the acrosomal zonadhesin (ZAN). Although systemic tolerance for ZAN is undetected in normal mice, when ZAN is exposed by unilateral vasectomy, it induces Ag-specific Treg-dependent tolerance in 7 days. Thus, when unilaterally vasectomized mice are immunized with the brain Ag or testis Ag, they develop an autoimmune disease in the brain but not in the testis. When unilaterally vasectomized mice are 60% Treg-depleted, they develop ZAN Ab and severe bilateral AO.

The rapid Treg response to the exposed sequestered sperm Ag resembles the response against microbial Ag, which may reduce collateral tissue damage and enhance infection chronicity (6). The newly appreciated sequel of murine vasectomy suggests that should receive further investigation, and that vasectomy should be avoided in rodent infection models. The fact that the sequestered sperm Ag are protected by Treg-dependent tolerance also raises new questions regarding the high immunogenicity of the “foreign” cancer/testis Ag (7) and whether vasectomy can lead to a predisposition to neoplasia.

Lustig et al. reviewed the overall functional anatomy of the testis and described the cells located in the testis interstitial space. They also describe in detail local mechanisms that operate in rat experimental AO (EAO). First, indoleamine 2,3-dioxygenase (IDO) produced by Sertoli cells and other cell types. The essential amino acid trytophan is deprived by IDO, and it leads to the production of kynurenine. Second, the cytokines IL6 and TNFα disrupt the BTB integrity and expose sperm Ag. Third, the dendritic cells (DC) present testis Ag to T cells and include Treg in draining the lymph node (LN). (8). Fourth, Treg spreads in the testis and regional LN during EAO, but they do not suppress T-cell response. Fifth, the activins promote fibrosis. Sixth, the testis macrophages increase.

The study from Bhushan et al. details the macrophages in two unique locations. Those in close contact with Leydig cells are yolk sac-derived and they self-renew. They are essential for Leydig cell function, including steroidogenesis that maintains BTB integrity, the hypothalamic–pituitary–gonadal axis, and spermatogenesis. Moreover, the macrophages co-localized with the spermatogonia are BM-derived and renewed from blood, and they support pre-meiotic germ cell development. The peri-tubular macrophages may also process and present endogenous and exogenous Ag to Ag-specific T cells locally and in the regional LN. The M2 macrophages in a normal testis express high levels of CD163 and IL10 and minimal levels of nitric acid and TNFα. They stimulate a Treg response and maintain local and systemic tolerance for testis Ag. However, when the testis is inflamed from AO, infections, or LPS exposure, the macrophages switch from M2 to M1. M1 macrophages are major histocompatibility complex class II+ (MHC Class II+), and they produce proinflammatory cytokines (TNFα and IL6) and chemokines. A new *in vitro* study by Fan et al. on the small Ca^2+^ binding protein S100A9 documented its effect on the immunosuppressive property of M2 macrophages. The process requires the activation of the P13kt pathway; and S100A9 and/or P13K inhibitors prevent the activation of M2 macrophages *in vitro*.

The immunology of the epididymis is quite different from the immunology of the testis, as described by Pleuger et al. and Zhao et al. Importantly, the proximal caput epididymis is structurally and functionally distinct from the distal cauda epididymis. The caput has a complex epithelial lining with intraepithelial lymphocytes surrounded by a network of dendritic cells and F4/80+ and CD11c+ macrophages. The latter projects cytoplasmic processes into the lumen to contact intraluminal cell contents (9). Interestingly, the caput lumen is the location where most cytoplasmic droplets (CD) are discarded by the epididymal sperm. Because CD shares a common origin with the RB, the caput is potentially a location of sperm Ag exposure. Two regulatory pathways are documented in the caput. The first is TGFβ (10). Mice deficient in dendritic cell-specific TGFβ receptor 2 spontaneously develop epididymitis and sperm Ab and accumulate Treg in the epididymis and testis. The second is IDO, activated by activin A, and is expressed in the caput (11). Mice deficient in IDO express pro-inflammatory cytokines and show epididymitis and abnormal sperm contents. In contrast, the cauda epididymis is lined by a simple epithelium, and it is more responsive to mechanical injury (vasectomy) and bacterial infections. The caudal interstitial macrophages express NFκB and toll-like receptors, mount innate responses against infectious agents, and are prone to granulomata and fibrosis.

The findings in this review suggest future directions for exploration that may better clarify the nature of human gonadal diseases. Said findings include how an autoimmune disease develops as well as what and how testis autoantigens are selected based on different mechanisms of autoimmunity, including those driven by infection. Future studies will benefit from active collaboration between research on local and systemic mechanisms.

## Author contributions

KT drafted the editorial, and DH and Y-GD contributed to the final submitted version. All authors contributed to the article and approved the submitted version.

## Conflict of interest

The authors declare that the research was conducted in the absence of any commercial or financial relationships that could be construed as a potential conflict of interest.

## Publisher’s note

All claims expressed in this article are solely those of the authors and do not necessarily represent those of their affiliated organizations, or those of the publisher, the editors and the reviewers. Any product that may be evaluated in this article, or claim that may be made by its manufacturer, is not guaranteed or endorsed by the publisher.
